# Pattern of recovery and outcomes of patient reported physical function and pain interference after ankle fusion: a retrospective cohort study

**DOI:** 10.1186/s41687-020-00203-y

**Published:** 2020-05-27

**Authors:** Jessica M. Kohring, Jeffrey R. Houck, Irvin Oh, Adolf S. Flemister, John P. Ketz, Judith F. Baumhauer

**Affiliations:** 1 Department of Orthopaedics, University of Rochester, 601 Elmwood Avenue, Box 665, Rochester, NY 14642 USA; 2 Department of Physical Therapy, George Fox University, Newberg, OR USA

**Keywords:** Ankle fusion, Patient reported outcomes, Function, Pain, PROMIS

## Abstract

**Background:**

Research on outcomes after ankle fusion focuses on basic activities of daily living, fusion rates, and gait parameters. Little has been reported on the patient’s perspective after surgery. The purpose of this study was to determine the change in patient reported physical function and pain interference after ankle fusion surgery to guide patient expectations and improve provider communication.

**Methods:**

This was a retrospective review of prospectively collected patient reported outcome measurement information system (PROMIS) data in 88 ankle arthrodesis procedures performed from May 2015 to March 2018. The PROMIS Physical function (PF) and pain interference (PI) measures were collected as routine care. Linear mixed models were used to assess differences at each follow-up point for PF and PI. Preoperative to last follow-up in the 120–365 day interval was assessed using analysis of variance. Outcomes included T-scores, z-scores, and PROMIS-Preference (PROPr) utility scores for PF and PI and the percentage of patients improving by at least 4 T-score points.

**Results:**

The linear mixed model analysis for PF after the 120–149 days, and for PI, after 90–119 days, indicated recovery plateaued at 39–40 for PF and 57–59 for PI T-scores. The change in the PI T-score was the greatest with a mean T-score improvement of − 5.4 (95% CI − 7.7 to − 3.1). The proportion of patients improving more than 4 points was 66.2% for either PF or PI or both. The change in utility T-scores for both PF (0.06, 95% CI 0.02 to 0.11) and PI (0.15, 95% CI 0.09 to 0.20) was significantly improved, however, only PI approached clinical significance.

**Conclusion:**

Average patients undergoing ankle fusion experience clinically meaningful improvement in pain more so than physical function. Average patient recovery showed progressive improvement in pain and function until the four-month postoperative time point. Traditional dogma states that recovery after an ankle fusion maximizes at a year, however based on the findings in this study, 4 months is a more accurate marker of recovery. A decline in function or an increase in pain after 4 months from surgery may help to predict nonunion and other complications after ankle arthrodesis.

**Level of evidence:**

Level II, prospective single cohort study.

## Introduction

Ankle arthrodesis remains the most commonly performed surgical procedure for end-stage osteoarthritis of the tibiotalar joint [27]. Overall, patients do quite well after ankle arthrodesis from a surgical standpoint, with high fusion rates between 83% to 99% [9, 15, 16, 21, 25, 28] and minimal complications [9, 21, 25, 28]. Recently, there has been an increasing emphasis on patient reported outcomes (PRO) to determine the success or failure after orthopaedic surgery. Functional outcome scores after undergoing ankle arthrodesis show improvement from pre- to postoperative scores. The majority of these results are based on questionnaires that focus on pain and disability in activities of daily living and on non-validated patient reported outcome measures like the American Orthopaedic Foot and Ankle Society (AOFAS) score [3, 5, 16, 17, 20, 26, 29]. These questionnaires focus on basic functional outcomes such as walking and stair-climbing and are specific to foot and ankle function, not to a patient’s general health status or ability level.

The success of a foot and ankle surgery in the eye of the patient is the ultimate goal of care. A recent study suggested that a majority of patients perceive their surgery as a success after a variety of different types of foot and ankle procedures [2]. However, this study was not specific to patients undergoing ankle fusion. Pre- and postoperative responses on Patient-Reported Outcomes Measurement Information System (PROMIS) symptom scales for patients undergoing ankle arthrodesis have not been reported. An advantage of using the PROMIS measures is that health related quality of life (HRQL) estimates can be determined from specific PROMIS domains such as physical function (PF) and pain interference (PI) [4]. Including HRQL improves interpretation because HRQL reflects the impact of health status (i.e. PF or PI) on quality of life [4]. In addition, the recovery “roadmap” based on patient reported outcomes after ankle fusion has not been previously described in the literature. This recovery roadmap can aid in assisting a patient’s understand temporal patterns of changes in pain and function after ankle fusion. Questions such as “when can I walk up stairs to go to bed rather than sleep on the couch after surgery?” or “when will the pain subside to the degree that I don’t need pain medications?” are relevant and important questions to answer to guide patients through their recovery. Additionally, follow-up has been based on a variety of time points with the final 1 year visit suggested as the timeframe that patients have maximized improvement. It is unknown if this is based on fact or dogma [4].

The purpose of this study was to determine the temporal change in patient reported outcome measures using the validated PROMIS [10–14, 19] T-score for patients undergoing ankle arthrodesis for primary ankle arthritis. We hypothesized that patients would experience a greater level of pain relief than improvement in physical function postoperatively. A second goal of this study was to determine the recovery pattern roadmap and duration of follow-up necessary for patients to maximize recovery after ankle fusion.

## Materials and methods

This study was performed at a single tertiary academic institution. Study approval was obtained through our center’s Global Institutional Review Board for patients with prospectively collected PROMIS data. Patients who underwent primary ankle arthrodesis between May 2015 and March 2018 were identified from a review of prospectively collected data. Patient reported PROMIS PF and PI T-scores were measured as part of the routine care via the PROMIS computer adaptive tests. Patient data was included if a preoperative PRO score and at least one postoperative PRO score were available at less than 365 after surgery. The following concomitant operative procedures were included: Achilles lengthening, gastrocnemius recession, or calcaneal osteotomy. Patients were excluded if they underwent other concomitant hindfoot or midfoot fusion procedures. All ankle arthrodesis procedures were performed by four fellowship-trained foot and ankle surgeons either open or arthroscopically, using screws alone or screws and a compression plate for fixation with or without bone graft augmentation. Patient demographics were collected for this subset of patients from a chart review.

### PROMIS outcome measures

The PROMIS PF and PI computer adaptive symptom scales were administered prior to a provider visit in the waiting room via an iPad. Standard instructions are provided by staff to patients prior to completing the PROMIS measures. The PROMIS PF measure indicates self-reported functioning of one’s upper extremities (dexterity), lower extremities (walking or mobility), and central regions (neck, back), as well as instrumental activities of daily living, such as running errands [23, 24]. The PROMIS PF T-score was used to determine the pre- and postoperative functional abilities of patients. The PROMIS PI (also known as “pain impact”) refers to the degree to which pain limits or interferes with an individuals’ physical, mental and social activities [1]. The PROMIS PI T-score was used to evaluate pre- and postoperative pain. Each scale is referenced to the U.S. population with 50 equal to the average of the US population and 10 points represents one standard deviation. For PROMIS PF, higher scores indicate better function. For PROMIS PI, lower scores indicate lower pain symptoms. Previously published data has indicated threshold T-scores for improvement in PF and PI after surgery as well as the minimally clinically important difference (MCID) change in post-operative PROMIS scores [6, 8, 12].

### Statistical analysis

PROMIS measures were collected preoperatively and then at regular time intervals for postoperative data. When multiple visits fell within the same time interval, the furthest follow-up date was used in the analysis. Linear mixed models were used to assess for differences in in PF and PI at each time point. A fixed model with maximum likelihood estimation was used to determine differences between each time interval (preoperative, 0–29 days, 30–59 days, 60–89 days, 90–119 days, 120–149 days, 150–179 days, 180–209 days, and greater than 210 days). The fit of the linear mixed model was assessed by evaluating the Akaike Information and Schwarz’s Bayesian Information Criteria to select the covariance structure that best fit these longitudinal measures within subject. The covariance types that were considered included compound symmetry (CS), CS heterogeneous, first order autoregressive (AR), AR unstructured, toeplitz and toeplitz heterogenous. From these model parameter estimates and the fitted covariance structure, differences between time points were assessed. The point at which the scores plateaued was used to determine the time interval to assess average overall effects for PF and PI. Analysis included 1) comparison of each time interval to pre-op and 2) comparing each time interval to the previous time interval.

Our secondary analysis used the last follow up in the 120 to 365 day window to determine MCID and utility scores associated with HRQL. The proportion of patients experiencing an MCID improvement was calculated from the preoperative to longest follow up in the 120 to 365 day interval data. Estimates of MCID vary, ranging from T-score improvement of approximately 4 to much larger values [6, 8, 12]. Minimal detectable change values are much stricter and therefore require much larger thresholds for meaningful change (> 12.6 T-score points) [12]. This study focused on capturing *ANY* potentially beneficial effects for these patients who have received an end stage procedure. Therefore, the proportion of patients experiencing an MCID improvement defined as a threshold of 4 T-score points were calculated for PF and PI. Estimates of the utility of a health state (PF or PI) are possible using the PROMIS-Preference (PROPr) scoring system [4]. Although a multi-attribute scoring is optimal, single attribute scoring functions are also useful when all 7 PROMIS domains used for multi-attribute scoring are not available [4]. Applying the single attribute scoring functions for PF and PI results in a utility score that varies from 0 to 1, where 0 is low utility and 1 high utility. Deviations lower than 1 are associated with lower health related quality of life. For patients with both pre-operative and follow up data in the 120 to 365 day interval, a two way repeated measures ANOVA was used to determine average improvement. Factors were time (preoperative and last follow up) and PROMIS measure (PF and PI). The significance of average preoperative to postoperative change in T-scores was determined using pairwise comparisons. To assess clinical relevance of aggregate differences, the estimated utility scores associated with HRQL were used.

## Results

Data were identified for 88 patients after ankle arthrodesis with at least one preoperative and one postoperative PROMIS PF and PI T-scores performed during the study timeframe. The mean age for this patient cohort at the time of surgery was 58.4 years (±15.0). There were 50 males and 38 females. There were PROMIS data available for a subset of 68 patients at pre-op and greater than 4 months after surgery (Table 1). The average follow-up after surgery was 232.3 days (range, 123 to 364). Data from the preoperative visit showed the average PI (T-score 63.2;) and PF (T-score 36.5;) symptoms were worse than one standard deviation (PI z-score 1.32 and PF z-score − 1.35) from the mean score of the United States population (T-score 50) (Table 1).

**Table 1:** Mean pre-op and follow up physical function and pain interference variables for patients with pre-op and follow up data greater than 120 days (*n* = 68)

Variable	Pre-Op Mean (SD)	Follow Up > 120 days Mean (SD)	Change (95% CI)	***p***-value
Days^a^	59.5 (53.5)	232.3 (79.0)	–	–
Physical Function	36.5 (6.1)	39.8 (7.3)	3.3(1.6 to 5.0)	< 0.01
Pain Interference	63.2 (7.2)	57.8 (8.3)	−5.4(− 7.7 to − 3.1)	< 0.01
Physical Function z –score	− 1.35 (0.61)	−1.02 (0.73)	0.33(0.16 to 0.50)	< 0.01
Physical Function HRQL^a^ Utility Estimate	0.55 (0.17)	0.61 (0.19)	0.06(0.02 to 0.11)	< 0.01
Pain Interference z-score	1.32 (0.72)	0.78 (0.83)	−0.54(− 0.77 to − 0.32)	< 0.01
Pain Interference HRQL^a^ Utility Estimate	0.61 (0.21)	0.75(0.19)	0.15(0.09 to 0.20)	< 0.01

*HRQL* health related quality of life, *CI* Confidence interval, *SD* standard deviation

^a^Days for Pre-op = number of days between evaluation and surgery; Days follow up = number of days from surgery to follow up

**Fig. 1:**
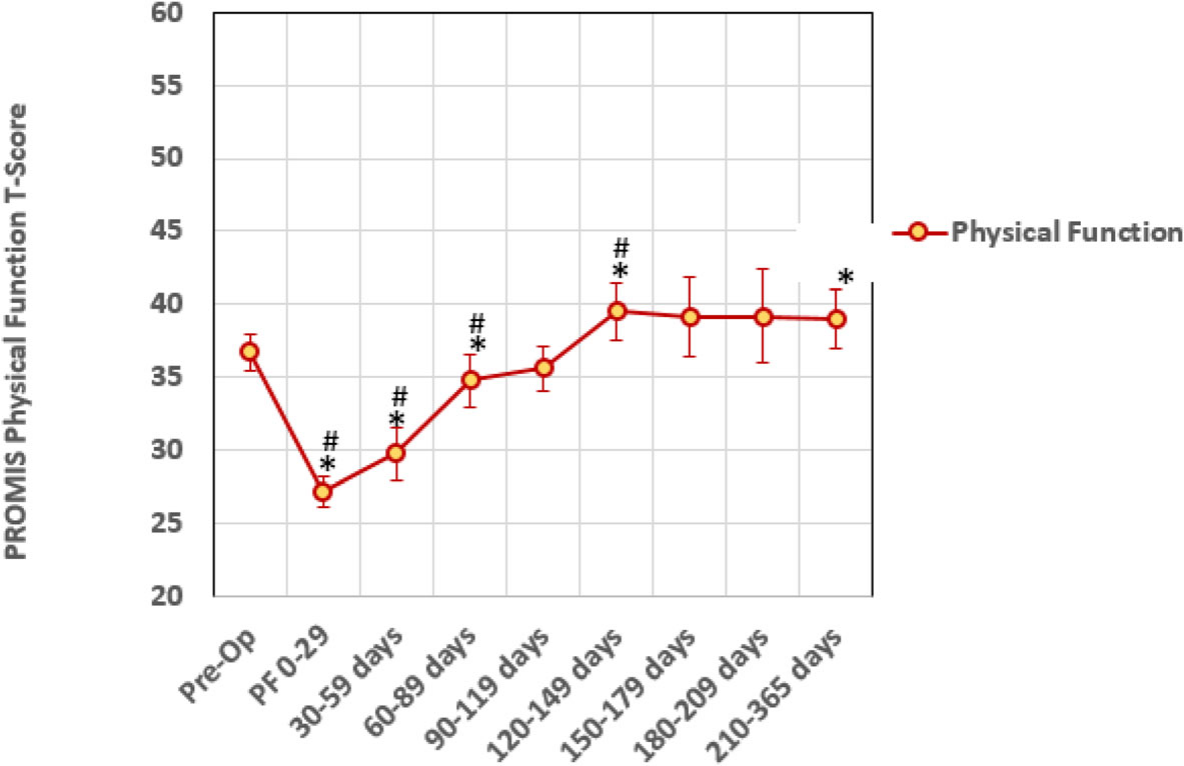
Recovery roadmap for PROMIS physical function T-scores for ankle arthrodesis patients. A lower T-score indicates improvement in symptoms. Error bars are the standard error of the mean for each time point. * = significance difference (*p* < 0.05) from pre-op to follow up time interval. # = significant difference(*p* < 0.05) from previous time point. Significance was determined using linear mixed model analysis (*n* = 88)

**Fig. 2:**
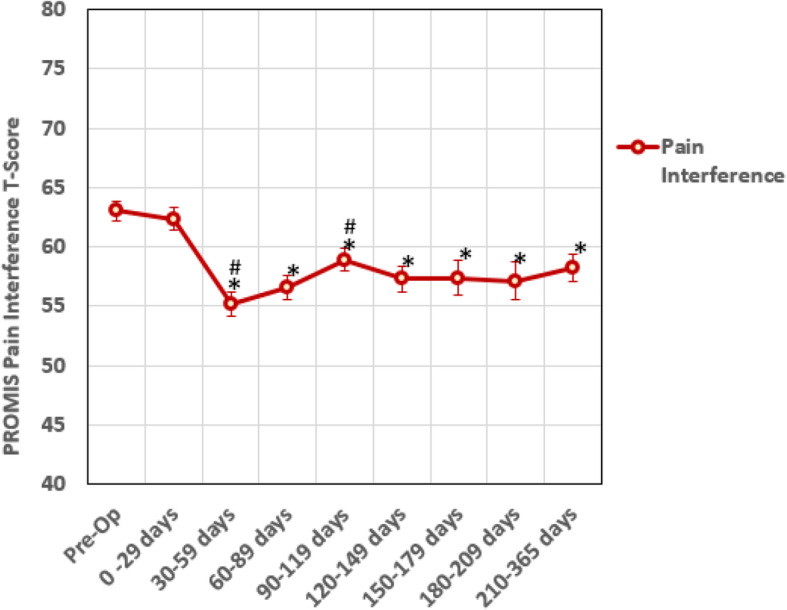
Recovery roadmap for PROMIS pain interference T-scores for ankle arthrodesis patients. A higher T-score indicates improvement in symptoms. Error bars are the standard error of the mean for each time point. * = significance difference (*p* < 0.05) from pre-op to follow up time interval. # = significant difference(*p* < 0.05) from previous time point. Significance was determined using linear mixed model analysis (*n* = 88)

The recovery roadmap for PF (Fig. 1) and PI (Fig. 2) outcomes for the 88 patients preoperatively and all available postoperative data at regular time intervals showed plateau points at less than 6 months. The linear mixed models analysis for PF (Table 2) showed that *after* the 120–149 day time interval, no time intervals were significantly different than the previous time point. When comparing time intervals to the preoperative time point, time intervals longer than 120–149 days showed marginal improvement in PF varying from 2.2 to 2.8 T-score points; two of which were statistically different than the preoperative time point. For the PI (Table 3), no time intervals were significantly different *after* the 90–119 day interval. When comparing time intervals to the preoperative time point, all time intervals except the 0–29 day interval showed significant improvement in PI varying from − 7.1 to − 4.2 T-score points. At the 120 to 149 day timeframe, the PF recovery roadmap showed a plateau in PF T-scores of approximately 40 (Table 4) with all data after this time point corresponding to minimal difficulty climbing stairs. Similarly, the PI T-scores of 57–59 (Table 4) plateaued at the 90 to 120 day timeframe corresponding to mild to moderate pain.

**Table 2:** Mean change across time for physical function from linear mixed model analysis (*n* = 88)

	Compared to PreOp Time Interval			Compared to Previous Time Interval		
Time Interval	Mean Change	95% Confidence Interval	***p***-value	Mean Change	95% Confidence Interval	***p***-value
0–29 days	−9.6	−11.1 to − 8.0	**< 0.01**	− 9.6	− 11.1to − 8.0	**< 0.01**
30–59 days	−6.9	− 8.5 to − 5.3	**< 0.01**	− 2.6	−4.3 to − 1.0	**< 0.01**
60–89 days	− 1.9	− 3.6 to − 0.2	**0.03**	5.0	3.3 to 6.8	**< 0.01**
90–119 days	− 1.1	− 2.9 to 0.75	0.25	0.8	−1.1 to 2.8	0.39
120–149 days	2.8	0.66 to 4.9	**0.01**	3.9	1.5 to 6.2	**< 0.01**
150–179	2.2	−0.4 to 4.7	0.10	−0.7	−3.7 to 2.3	0.66
180–209 days	2.5	−0.4 to 5.3	0.09	0.4	−3.2 to 3.9	0.85
210–365 days	2.2	0.2 to 4.2	**0.03**	−0.3	−3.4 to 2.9	0.83

**Table 3:** Mean change across time for pain interference from linear mixed model analysis (*n* = 88)

	Compared to PreOp Time Interval			Compared to Previous Time Interval		
Time Interval	Mean Change	95% Confidence Interval	***p***-value	Mean Change	95% Confidence Interval	***p***-value
0–29 days	−0.7	−1.2 to 2.5	0.48	−0.7	− 1.2 to 2.5	0.48
30–59 days	−7.9	−9.8 to −6.0	**< 0.01**	− 7.2	− 9.1 to − 5.2	**< 0.01**
60–89 days	−6.4	−8.4 to −4.5	**< 0.01**	1.5	− 0.6 to 3.5	0.17
90–119 days	− 4.2	− 6.3 to − 2.0	**< 0.01**	2.3	0.0 to 4.5	**0.05**
120–149 days	−5.7	−8.2 to − 3.1	**< 0.01**	− 1.5	−4.3 to 1.23	0.29
150–179 days	− 5.7	−8.7 to − 2.7	**< 0.01**	− 0.0	− 3.6 to 3.5	0.98
180–209 days	− 5.9	− 9.3 to − 2.5	**< 0.01**	− 0.2	−4.4 to 4.0	0.94
210–365 days	−4.8	− 7.2 to − 2.4	**< 0.01**	1.1	−2.7 to 4.8	0.58

**Table 4:** Mean and standard error of the mean^a^ (SE) for pain interference and physical function for each time point using all participants data (*n* = 88)

Time Interval	n	Pain Interference (SE)	Physical Function (SE)
Pre-Op	88	63.1 (0.9)	36.7 (0.7)
0–29 days	79	62.4 (0.9)	27.1 (0.7)
30–59 days	72	55.2 (0.9)	29.8 (0.8)
60–89 days	65	56.6 (1.0)	34.8 (0.8)
90–119 days	50	58.9 (1.1)	35.6 (0.9)
120–149 days	32	57.4 (1.3)	39.6 (1.0)
150–179 days	21	57.3 (1.5)	38.8 (1.2)
180–209 days	16	57.2 (1.7)	39.2 (1.4)
210–365 days	37	58.2 (1.2)	38.9 (1.0)

^a^Means = estimated marginal means from the linear mixed model abalysis

The two way ANOVA results (*n* = 68) showed improved average PROMIS outcomes from preoperative to postoperative follow-up (120 to 365 days) (Table 1). The change in the PI T-score was the greatest with a significant (*p* < 0.001) mean T-score decrease of − 5.4 (95% CI − 7.7 to − 3.1). This resulted in a PI T-score of 57.8 (±7.3) at follow-up, approximately 0.8 standard deviations (z-score = 0.78) worse than the mean US normative data. The PROMIS PF T-score showed smaller improvement with a significant mean T-score change of 3.3 (95% CI 1.6 to 5.0). The mean postoperative PF T-score was 39.8 (±7.3), one standard deviation below the average for the US average (z-score = − 1.02 (0.73). The mean postoperative PF T-score showed significant improvement from the mean preoperative T-score of 36.5 (±6.1) (*p* < 0.01). The proportion of patients improving more than a MCID was 47.1% for PF, 55.9% for PI and 66.2% for either PF or PI or both. The change in utility scores for both PF (0.06, 95% CI 0.02 to 011) and PI (0.15, 95% CI 0.09 to 0.20) showed a significant statistical improvement in HRQL with the follow-up utility scores for PF (0.61 (±0.19)) and PI (0.75 (±0.19)) demonstrating some continued pain and functional impairment from ideal health (1.0).

## Discussion

Our findings in this study showed significant preoperative pain and physical function impairment relative to the US population mean data for patients presenting with advanced stage ankle arthritis and who subsequently underwent ankle arthrodesis based on PROMIS PF and PI assessments. At the preoperative visit, the PROMIS PF and PI T-scores were consistent with studies suggesting that surgical intervention would likely lead to improved patient reported outcomes postoperatively [6, 8, 12]. The average postoperative change in PROMIS measures were modest resulting in improvements in HRQL utility scores of 0.06 for PF and 0.15 for PI at 120 to 365 days after ankle fusion. The postoperative change in PI T-score showed the greatest improvement compared to PF PROMIS symptom scales. The percentage of patients experiencing an MCID was less for PF (47.1%) than PI (55.9%), however, 66.2% of patients experienced an MCID change in one or both of the measures. The PI T-score indicated that the average patient after surgical follow up was not pain-free, however experienced improvement in HRQL. Based on these results, patients appear to gain the most benefit in pain reduction after ankle arthrodesis. Physical function outcomes are also improved, but to a lesser extent. If the patient’s goal is to improve in global physical function with the treatment of end-stage ankle arthritis, an arthrodesis may not be the best surgical option.

Previous studies have noted an association between satisfaction and pain after ankle arthrodesis. Thomas et al. found high patient satisfaction and reliable pain relief in 26 patients undergoing ankle arthrodesis for ankle arthritis. However this patient cohort showed ongoing and significant decrease in hindfoot function and gait abnormalities after surgery as compared to a control group [26]. Similar results have been replicated in multiple studies showing reliable improvement in pain and high satisfaction rates, but with less improvement in function [5, 29]. In light of the results in this study and previous reports, patients should be educated preoperatively that they are likely to experience a greater improvement in pain relief, rather than function to manage postoperative expectations appropriately. Additionally, patients should be counseled that they will not be pain-free after ankle fusion, but will experience a significant decrease in their pain level during daily activities.

PROMIS physical function T-scores have been linked to functional tasks called a snapshot [27]. In our study, preoperatively the mean PF T-score was 36.5, based on the T-score snapshot for this value, patients had difficulty in walking short distances, going up and down less than five flights of stairs, and carrying heavy objects going up stairs. At greater than 4 months after surgery, the mean PF T-score improved to 39.8. A PROMIS PF T-score of 40 corresponds to the ability to walk briskly for at least 20 min with no break, having little difficulty going up and down at least five flights of stairs, and the ability to carry heavy objects upstairs, based on the T-score snapshot [7]. Pinsker et al. showed that most patients reported positive outcomes after undergoing ankle arthrodesis or arthroplasty for end-stage ankle arthritis, but the majority did not experience resolution of all symptoms. These authors suggested that patients achieved improvement in symptoms and limitations at a level of residual deficits that they were able to cope with and they felt this was a satisfactory outcome [22]. Kerkhoff et al. showed similar results in their study assessing functional outcomes and pain after undergoing ankle arthrodesis. At a mean follow-up of 8 years, patients’ Foot Function Index mean pain score significantly improved from 49.5 (+/− 18.9) preoperatively to 20.0 (+/− 25.1) postoperatively. Physical function, including sports participation diminished after undergoing ankle arthrodesis. Patients remained active with decreased pain after surgery, but participated in less demanding activities [18]. In-line with our study, patients can expect to achieve improvement in activities of daily living, but not complete resolution of all limitations as they are likely to continue to experience some pain and functional limitations even after an ankle fusion for end-stage ankle arthritis. The utility scores suggest modest improvement in HRQL consistent with interpretation of the T-scores. The PRO recovery roadmap, T-scores, and utility scores help to answer patient questions and align patient expectations with surgery.

Our study also identified a recovery curve or roadmap for the average improvement in physical function and pain interference outcome measures after undergoing ankle fusion. Due to the fact that each time interval does not include all participants, these recovery curves are useful to assess average not individual improvement over time. The average data shows that at approximately 4 months after surgery, improvement in physical function and pain plateau with minimal changes after this time point. This suggests that on average patients experience the greatest change in function and pain during the first 4 months after surgery. This is contrary to the dogma requiring 1 year follow-up to maximize surgical improvement stated by insurance companies and worker compensation. It may be reasonable, and perhaps preferable, to have patients to return to clinic on an as needed basis after this time point if they show an appropriate level of improvement which would lead to saving healthcare dollars for the patients and the system. Patient reported outcome measures allow a provider to determine a response to treatment and follow an average pattern of recovery. After ankle arthrodesis, patients in our study experienced a progressive improvement during the first 4 months after surgery. Alternative patterns demonstrating a decline may help to predict complications with surgery.

In recent years, there has been an increasing emphasis on understanding the patient’s perspective of outcomes after an orthopaedic surgery or procedure. The majority of previous studies looking at outcomes after treatment for end-stage ankle arthritis have used non-validated patient questionnaires to quantify changes in pain and physical function. One of the strengths of this study was based on the use of the validated PROMIS outcome measures to assess pre- and postoperative physical function and pain for ankle fusion patients.

There are several limitations with this study, including smaller numbers of patients and consistent preoperative and follow-up time points. The preoperative and follow-up data are inconsistent because of practical requirements for surgeons to balance tracking patient status, the need for improved efficiency (patient and provider time) and lower costs to patients and the healthcare system. Ankle arthrodesis is an end stage elective procedure, it is not uncommon for the pre-operative visit when the decision for surgery is made to occur several weeks prior to surgery. So although the closest data to the surgical date was used in the analysis, the pre-operative visit varies from the surgical date. Similarly, although follow ups are planned during recovery, many practical issues make these follow up time points inconsistent. This explains the relatively small number of follow-up PROMIS data available at greater than 6 months after surgery. Standardized longer-term follow-up and a larger patient cohort will improve power and clinical significance for future similar studies, however this may not change the overall initial course of treatment. Further, the MCID values are dependent on which method is used to calculate MCID. And, should reflect values that are higher than minimal clinically detectable change. Finally, estimates of the HRQL utility score are based on single attribute functions, multi-attribute scoring may modify these findings by including other dimensions of HRQL [4].

## Conclusion

This study demonstrates, on average, patients experience a clinically meaningful improvement in pain and a marginal improvement in physical function after undergoing ankle fusion. In the context of HRQL it is likely that the improvement in pain has the greater benefit to these patients after ankle arthrodesis. The results of this study and the roadmap for recovery can assist the orthopaedic surgeon in preoperative counseling and postoperative expectation management for patients seeking treatment for end-stage ankle arthritis.

## Data Availability

Data sharing is not applicable to this article as no datasets were generated or analysed during the current study.

## Abbreviations

HRQL: Health Related Quality of Life
PRO: Patient Reported Outcome
PF: Physical Function
PI: Pain Interference
PROMIS: Patient-Reported Outcomes Measurement Information System
MCID: Minimally Clinically Important Difference
